# Standardization of an antimicrobial resistance surveillance network through data management

**DOI:** 10.3389/fcimb.2024.1411145

**Published:** 2024-07-29

**Authors:** Hyunji Kim, Jeong Su Park, Dokyun Kim, Hee Jung Kim, Jeong Hwan Shin, Young Ah Kim, Young Uh, Soo Hyun Kim, Jong Hee Shin, Seok Hoon Jeong, Kyoung Un Park

**Affiliations:** ^1^ Department of Laboratory Medicine, Seoul National University Bundang Hospital and Seoul National University College of Medicine, Seoul, Republic of Korea; ^2^ Department of Laboratory Medicine and Research Institute of Bacterial Resistance, Yonsei University College of Medicine, Seoul, Republic of Korea; ^3^ Department of Laboratory Medicine and Paik Institute for Clinical Research, Inje University College of Medicine, Busan, Republic of Korea; ^4^ Department of Laboratory Medicine, National Health Insurance Service Ilsan Hospital, Goyang, Republic of Korea; ^5^ Department of Laboratory Medicine, Yonsei University Wonju College of Medicine, Wonju, Republic of Korea; ^6^ Department of Laboratory Medicine, Chonnam National University Medical School, Gwangju, Republic of Korea

**Keywords:** antimicrobial resistance, Kor-GLASS, quality control center, IPT, EQA

## Abstract

**Introduction:**

The rapid spread of COVID-19 worldwide within 2 months demonstrated the vulnerability of the world’s population to infectious diseases. In 2015, the Global Antimicrobial Resistance and Use Surveillance System (GLASS) was launched to combat antimicrobial resistance (AMR). However, there has been no comprehensive assessment of the decade-long global battle against AMR based on GLASS data.

**Methods:**

South Korea established Kor-GLASS (Korean-GLASS) to proactively monitor data quality and enable international collaborations. A unique feature of Kor-GLASS is the quality control center (QCC), which uses network hubs and ensures standardized, high-quality data through interlaboratory proficiency testing (IPT) and external quality assessment (EQA). In addition, the QCC multifaceted endeavors for integrated data quality management.

**Results:**

Since 2020, high-quality AMR data have indicated fluctuating antibiotic resistance rates in South Korea. This trend does not align with the decrease in antibiotic usage seen in humans but coincides with non-human antibiotic sales, indicating a need for greater monitoring of non-human antibiotic resistance. Comprehensive and robust management taking account of the intricate interplay among humans, animals, and the environment is essential. Kor-GLASS has been expanded into a “One Health” multiagency collaborative initiative.

**Discussion:**

Although a standardized solution is not suitable for all countries, it must align with the local context and international standards. A centralized top-down management structure such as that of the QCC is essential to ensure continuous data quality coordination. Sustained efforts and surveillance systems are crucial for monitoring and managing AMR and safeguarding human health.

## Introduction

In 2014, the O’Neill report highlighted the global threat posed by antimicrobial resistance (AMR) (O’Neill, 2014). Subsequently, in 2015, during the 68th World Health Assembly, the Global Antimicrobial Resistance and Use Surveillance System (GLASS) was established as a worldwide action plan by the World Health Organization (WHO) (Organization WHO, 2014). The rapid and widespread outbreak of COVID-19 in 2019, affecting over 200 countries within a span of two months, demonstrated the vulnerability of the world’s population to infectious diseases (Lim, 2021). This experience underscores the importance of robust surveillance systems for managing public health threats.

South Korea has been actively involved in AMR management since 1997, prior to the establishment of GLASS (**Supplementary Figure S1**, **Supplementary Table S1**). In 2016, South Korea launched the Korean Global Antimicrobial Resistance Surveillance System (Kor-GLASS), which is based on the GLASS platform but incorporates additional features aimed at enhancing data quality and management. Kor-GLASS operates under a “One Health” approach (KDCA, 2017-2021; Lee et al., 2018; Liu et al., 2019), which integrates human, animal, and environmental health sectors to comprehensively address AMR.

The primary objective of Kor-GLASS is to provide standardized, high-quality data that can be used for further analysis, interpretation, and actions by various stakeholders. The system includes key components such as external quality assessment (EQA) and interlaboratory proficiency testing (IPT) to ensure data quality. The Quality Control Center (QCC) plays a central role in managing these components and maintaining the integrity of the data collected.

This study aims to provide a detailed overview of the Kor-GLASS system, highlighting its unique features, innovative approaches, and contributions to the global fight against AMR. By comparing Kor-GLASS with other national AMR surveillance systems, this paper seeks to emphasize the system’s unique contributions and improvements, and to discuss its potential impact on AMR management.

## Methods

### Network organization of Kor-GLASS

**Figure 1:**
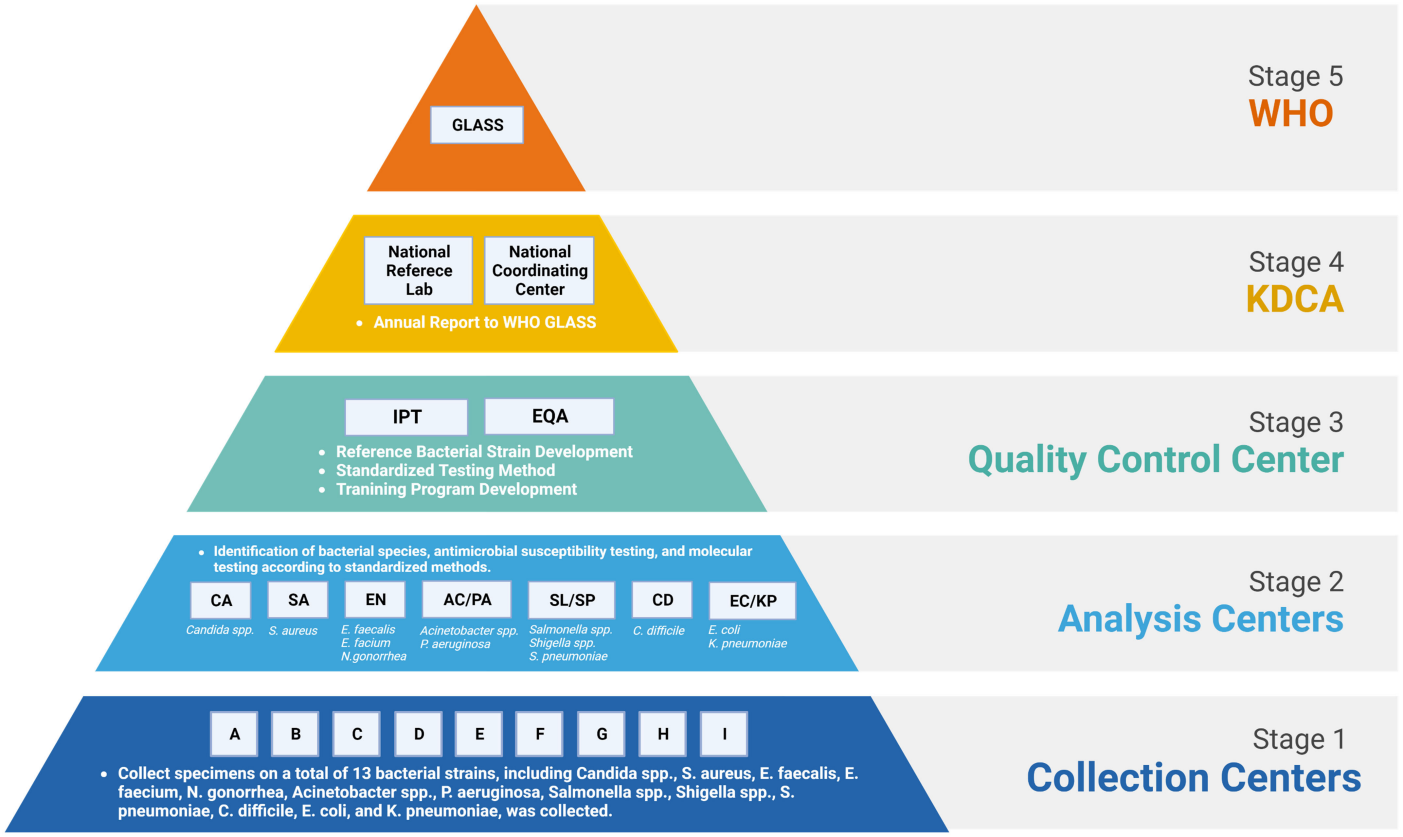
Organizational structure, relationships, and government and international institutions within Kor-GLASS. WHO, World Health Organization; GLASS, Global Antimicrobial Resistance and Use Surveillance System; KDCA, Korea Disease Control and Prevention Agency; IPT, interlaboratory proficiency testing; EQA, external quality assessment. Figures were created with BioRender.

**Figure 2:**
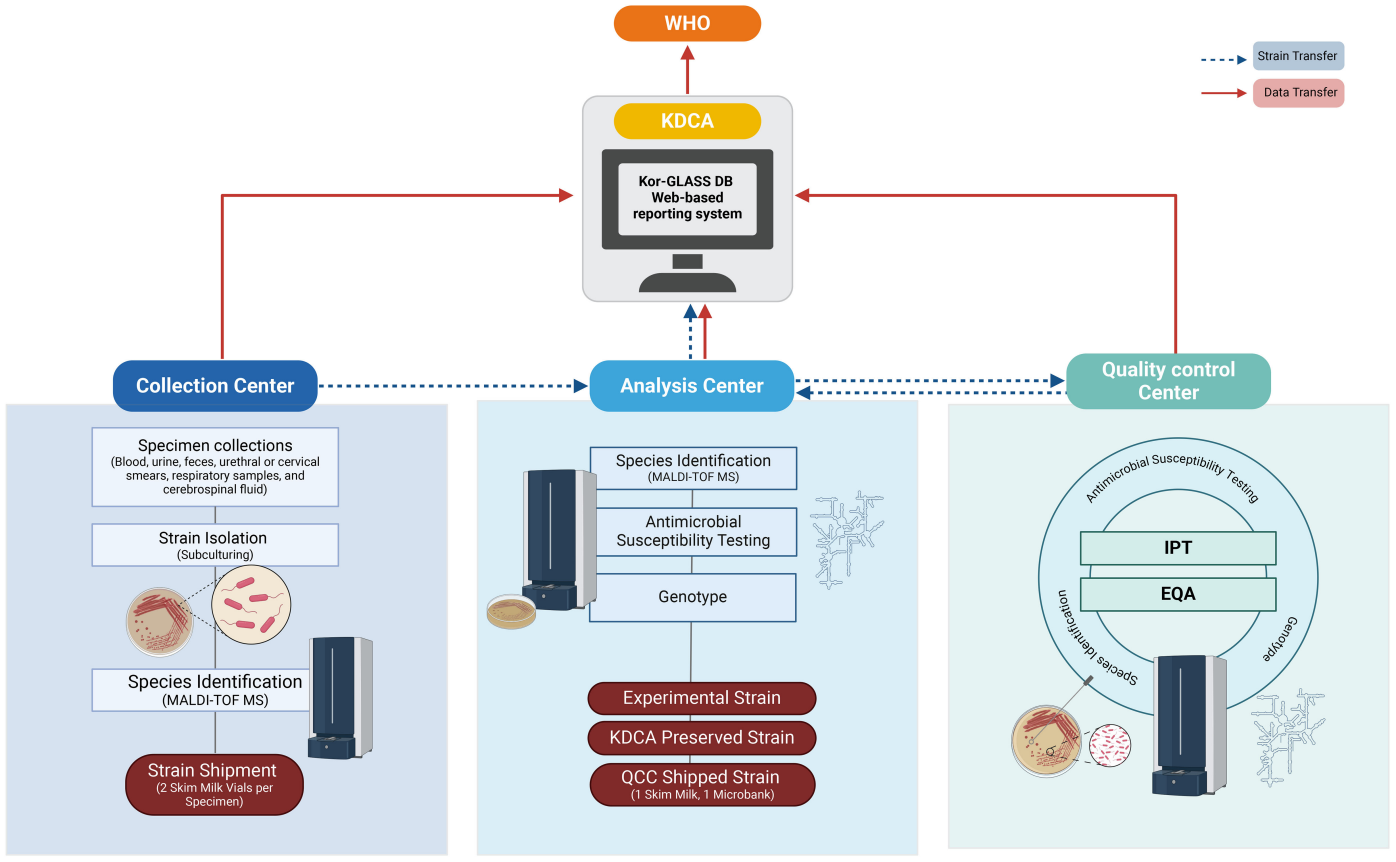
Flowchart of Strain Collection and Data Flow. The flowchart illustrates the process of strain collection and data flow, following two primary pathways: one from the collection center to either the analysis center or the quality control center, covering processes from identification to test management, and another from the analysis center to KDCA for strain preservation. Data collected by each center is uploaded to the Kor-GLASS database (https://is.kdca.go.kr/) in the designated format. This data is managed by KDCA, which reports the annual results to WHO in the GLASS format, ensuring precise tracking and reporting for high data integrity and quality control. Figures were created with BioRender.

Kor-GLASS was established to collect standardized AMR surveillance data in accordance with the GLASS guidelines, toward the construction of a global database. This initiative is steered by the Korea Disease Control and Prevention Agency (KDCA), a government agency. To ensure advancement of the AMR surveillance system, the government has appointed experts within a hierarchical framework. Kor-GLASS is based on four principles: representativeness, specialization, harmonization, and localization (Lee et al., 2018). The system is divided into five stages (**Figure 1**), systematically integrating specimen collection, data generation, and data quality assessment. Testing methods and results are standardized across all centers, and these uniform results are uploaded to the Kor-GLASS database (https://is.kdca.go.kr/). The operational framework for each hub ensures a consistent flow of specimens and data, with detailed processes outlined in **Figure 2** and **Supplementary Table S2**.

### Quality control center

A distinctive feature of Kor-GLASS is the establishment of a national-level Quality Control Center (QCC), which is independent of the GLASS system. This centralized QCC bridges gaps between network hubs, ensuring the generation of standardized, high-quality AMR surveillance data through unified criteria, and facilitating rapid improvements and issue resolution across the system.

**Figure 3:**
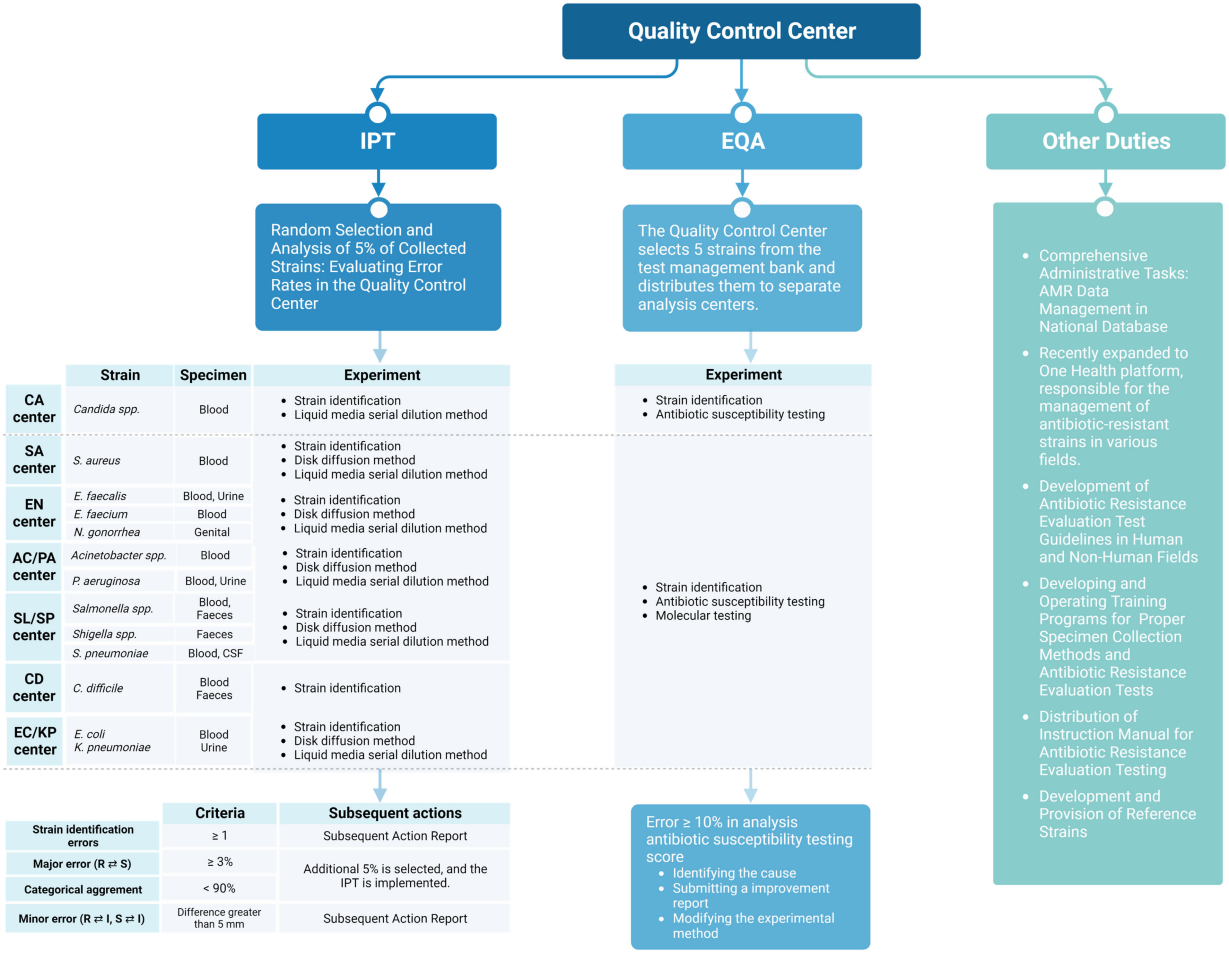
Role of the quality control center in antibiotic-resistant surveillance in Korea. Figures were created with BioRender.

Designated by the KDCA, the QCC operates independently of collection and analysis centers. It is a tertiary hospital laboratory with expertise in analysis, staffed with a dedicated on-site microbiology expert, and holds ISO 9001:2015 certification, ensuring adherence to stringent international standards. The QCC standardizes processes among analysis centers and manages the collected data, striving for network-wide consistency (**Figure 3**).

Additionally, the QCC ensures rigorous data quality management through IPT and EQA. Unlike other national AMR surveillance systems in countries such as the United States (Robillard et al., 2024), the United Kingdom (Bennani et al., 2021), and Australia (Coombs et al., 2024), the centralized QCC of Kor-GLASS operates autonomously, maintaining high standards across multiple centers.

#### Initial integrated data quality management: IPT and EQA

Monitoring of the seven analysis centers responsible for generating species-specific AMR data under the guidance of the QCC is essential for integrated data quality management. Antibiotic susceptibility testing (AST) is performed using disk diffusion or broth microdilution methods in accordance with the recommendations of the Clinical Laboratory Standard Institute and the European Committee on Antimicrobial Susceptibility Testing (Matuschek et al., 2014; Wayne and Clinical and Laboratory Standards Institute, 2016). For data quality management, the QCC has implemented bidirectional management through IPT and EQA, ensuring comprehensive data quality control.

IPT is typically conducted on a monthly basis for samples delivered from analysis centers to the QCC. The QCC evaluates the error rate by performing AST on randomly selected isolates (5% for all species) from the strains collected from each analysis center. Errors are categorized as major, involving misclassification of resistant strains as susceptible or vice versa, or minor, involving intermediate classifications. The accepted threshold for major errors is < 3%, and the categorical agreement for the classification of susceptible, intermediate, and resistant strains should be > 90%. In cases where major errors exceed 3% or categorical agreement falls below 90%, an additional 5% of randomly selected strains for the specified period are tested at the QCC. Causes of errors are investigated, and corrective actions are implemented.

EQA is conducted on a quarterly basis and involves the distribution of fully identified strains from the QCC to each analysis center. Five strains are selected from the QCC strain bank, and analysis centers undertake testing within a predefined scope, encompassing tasks such as species identification and AST. The QCC assesses the data generated by the analysis centers, presenting the results as percentages, with a weight of 5 assigned to the degree of agreement with the identification results. If the error rate exceeds 10%, causes of the errors are investigated, and corrective actions are implemented.

#### Enhanced data quality management

The AMR data provided by the seven analysis center hubs, which conform to uniform testing criteria and quality standards, are aggregated within the Disease Control and Prevention Integrated Management System database, which is managed by the KDCA (Kor-GLASS database: https://is.kdca.go.kr/). Access to this database is restricted to designated personnel within the Kor-GLASS network hubs. The QCC uploads findings from its oversight activities, ensuring high-quality national AMR data are reported to the WHO annually through the KDCA. Furthermore, comprehensive AMR management data are disclosed transparently and are accessible for public scrutiny via the KDCA platform (https://nih.go.kr/nohas/common/main.do).

#### Multifaceted endeavors for data quality management: training and education

To minimize errors made during specimen collection and testing, the QCC provides ongoing education and develops guidelines for standardized collection methods. Biannual visits to participating laboratories are conducted to assess infrastructure and techniques, followed by training to address identified issues. The QCC also educates on updated molecular microbiology testing techniques, which are incorporated into standardized experimental guidelines.

For new participant laboratories, it evaluates adherence to guidelines, provides the necessary training, and conducts EQA testing. The QCC meticulously characterized the phenotypic and genetic resistance profiles for the scrutiny of reference strains. Subsequently, QCC leverages preserved reference strains for ongoing research endeavors and proficiency assessments, such as EQA, among laboratories involved in the management of resistant bacteria.

This process involves the use of matrix-assisted laser desorption/ionization time-of-flight mass spectrometry (MALDI-TOF MS) and 16S rRNA sequencing techniques for species identification. In certain challenging cases, Acinetobacter strains are identified through additional sequencing of the *nuc* and *rpoB* genes. *SCCmec* typing and analysis of *Staphylococcus aureus* strains involves examining the *mecA* and *SCCmec* genes using 18 primers. Additional tests include *TSST-1* gene identification, analysis of *eta* and *etb* genes, multilocus sequence typing, spa typing, and whole-genome analysis using Pacific Biosciences equipment.

To develop strains for extended-spectrum beta-lactamase or carbapenemase proficiency testing, several genes are evaluated. In particular, *TEM, SHV*, and *CTX* genes are examined for extended-spectrum beta-lactamases, while *CMY, DHA*, and *CIT* genes are tested for plasmid-mediated *AmpC* β-lactamases. For carbapenemases, the *KPC, GES, VIM, IMP*, and *NDM* genes are analyzed. Additionally, *mcr-1* PCR and sequence analysis, along with multilocus sequence typing, are performed.

## Results

### Outcomes of integrated data quality management by the QCC

#### IPT implementation and findings

IPT is the most important responsibility of the QCC because it ensures quality assurance and standardization of data generated by the analysis centers. The frequency of IPT implementation varies across centers depending on the number of collected specimens. Between 2020 and 2021, the following specimens underwent AST testing and analysis of species-specific bacterial-antimicrobial combinations through IPT:

− For *E. coli*, 1,464 of 19,568 specimens underwent AST, resulting in the testing of 29,500 bacterial-antimicrobial combinations.

− For *Klebsiella pneumonia*, 364 of 4,608 specimens underwent AST, resulting in the testing of 6,973 bacterial-antimicrobial combinations.

− For *S. aureus*, 156 of 1,506 specimens underwent AST, resulting in the testing of 2,214 bacterial-antimicrobial combinations.

− For *Enterococcus faecium*, 94 of 761 specimens underwent AST, resulting in the testing of 1,119 bacterial-antimicrobial combinations.

− For *Enterococcus faecalis*, 62 of 447 specimens underwent AST, resulting in the testing of 802 bacterial-antimicrobial combinations.

− For *Acinetobacter baumannii*, 103 of 412 specimens underwent AST, resulting in the testing of 1,468 bacterial-antimicrobial combinations.

− For *Pseudomonas aeruginosa*, 69 of 442 specimens underwent AST, resulting in the testing of 925 bacterial-antimicrobial combinations.

**Figure 4:**
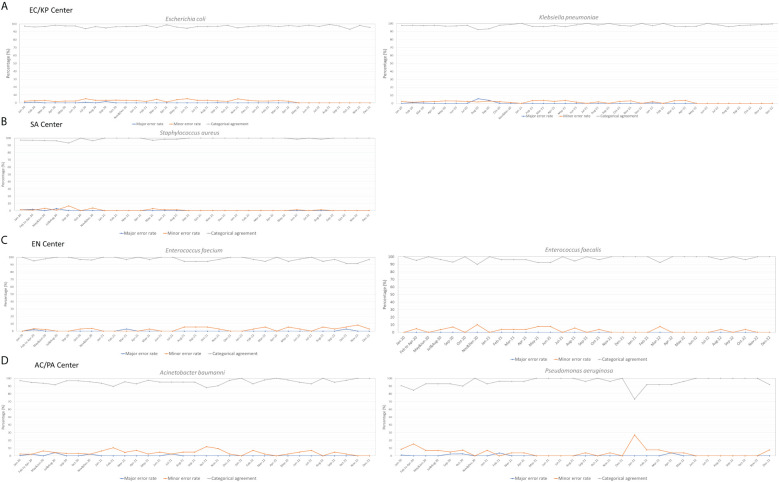
Three-Year Management Outcomes of the Quality Control Center: Ensuring Consistent Data Quality in Each Analysis Center. **(A)** EC/KP (Escherichia coli/Klebsiella pneumoniae) - Management outcomes of Escherichia coli and Klebsiella pneumoniae in the center; **(B)** SA (Staphylococcus aureus) - Management outcomes of Staphylococcus aureus in the center; **(C)** EN (Enterococcus) - Management outcomes of Enterococcus faecalis and Enterococcus faecium in the center; **(D)** AC/PA (Acinetobacter spp./Pseudomonas aeruginosa) - Management outcomes of Acinetobacter spp. and Pseudomonas aeruginosa in the center.

During this period, no species identification errors occurred in any of the centers. As depicted in **Figure 4**, the majority of bacterial species were subjected to monthly IPT. The AST results demonstrated categorical agreement within 90%, and no instances where major error rates exceeded 10%. However, some analysis centers had minor error rates > 10%, resulting in categorical agreement < 90%. In particular, minor error rates > 10% were noted in the results for *A. baumanni* in February and October 2021, as well as for *P. aeruginosa* in January 2022. These errors were attributed to inaccurate in determination of the size of inhibition zones according to EUCAST guidelines (EUCAST, 2024); subsequent education and retesting were performed to rectify these errors (See **Supplementary Figure S2** for error photos in Disk diffusion test across various strains.). In subsequent IPTs, the error rate had stabilized within an acceptable range, confirming the production of consistently high-quality data.

For centers with a lower volume of collected specimens, including SL/SP, CD, and CA centers where IPT procedures were performed every 3 months, the numbers of specimens that underwent IPT between 2020 and 2021 were as follows: 63 for *Candida* spp., 54 for *Clostridium difficile*, 41 for Salmonella spp., and 20 for *Streptococcus pneumoniae*. Notably, no errors or major discrepancies were observed in species identification among these specimens. For *Candida* spp., minor error rates were consistently low (average of 0.18%). Similarly, *Salmonella* spp. displayed a modest average error rate of 1.0%. However, *S. pneumoniae* showed a slight increase in minor identification errors during the initial half of 2021, with an average rate of 10.5%.

#### EQA performance and data quality

EQA is conducted quarterly and involves the distribution of fully identified strains from the QCC to each analysis center. The results of the EQA are presented as percentages. The error rates for all tested species were consistently low, indicating reliable data quality.

#### AMR trends in South Korea

**Figure 5:**
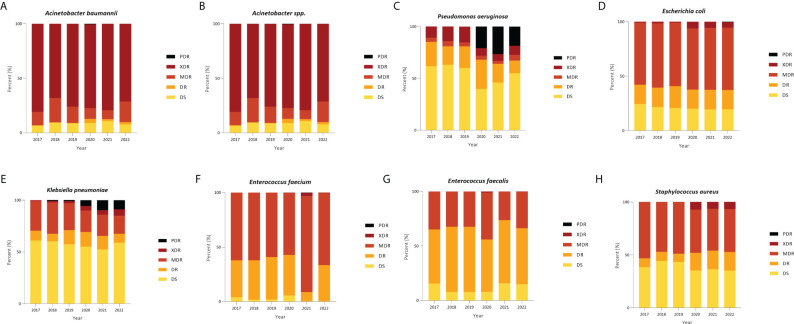
Distribution of categories of resistance among strains in blood. **(A)** *Acinetobacter baumannii*, **(B)** *Acinetobacter spp.*, **(C)** *Pseudomonas aeruginosa*, **(D)** *Escherichia coli*, **(E)** *Klebsiella pneumoniae*, **(F)** *Enterococcus faecium*, **(G)** *Enterococcus faecalis*, and **(H)** *Staphylococcus aureus*. PDR, pan drug resistance; XDR, extensive drug resistance; MDR, multi-drug resistance; DR, drug resistance; DS, drug-sensitive.

A comprehensive analysis of AMR fluctuations in South Korea revealed distinct patterns among various strains. High levels of AMR were observed in critical priority pathogens (Organization WHO, 2017), including *A. baumannii, P. aeruginosa*, and multiple Enterobacteriaceae (including *K. pneumoniae, E. coli*, Serratia, and Proteus), indicating the need for continuous monitoring and management (**Figure 5**). Notably, the detection rate of extensively drug-resistant *P. aeruginosa* surged from 20.87% in 2020 to 26.61% in 2021, remaining high at 18.63% in 2022. Similarly, the proportion of extensively drug-resistant *K. pneumoniae* increased from 5.75% in 2020 to 9.72% in 2021, slightly decreasing to 8.87% in 2022. Considering that Kor-GLASS gathers data from medium to large hospitals with 500-1000 beds, the sudden surge in antibiotic resistance witnessed in 2021 cannot dismiss the potential for a temporary shift linked to COVID-19 in the respiratory intensive care units (La et al., 2022).

#### Expansion into one health initiative

High-quality AMR data revealed that the AMR rate in Korea displayed marked fluctuations. Conversely, data from the Health Insurance Review and Assessment Service (HIRA, 2017-2021) indicated a gradual decline in antibiotic utilization in hospitals and pharmacies over the years. This decline is measured in terms of the daily dose per 1,000 Inhabitants per day (DID), which was 23.65 in 2017, 26.92 in 2018, 26.51 in 2019, 20.93 in 2020, and 19.58 in 2021. In contrast to these declining trends, Kor-GLASS verification data revealed fluctuating patterns, rather than the expected decrease in antibiotic resistance rates.

The antibiotic sales volume data provided by the Animal and Plant Quarantine Agency (2017-2021) revealed a fluctuating pattern: 1,003,678 kg in 2017, 960,663 kg in 2018, 903,476 kg in 2019, 894,999 kg in 2020, and 1,035,850 kg in 2021. The observed fluctuations in AMR rates within the Kor-GLASS data can be attributed to the increasing antibiotic use in non-human sectors.

**Figure 6:**
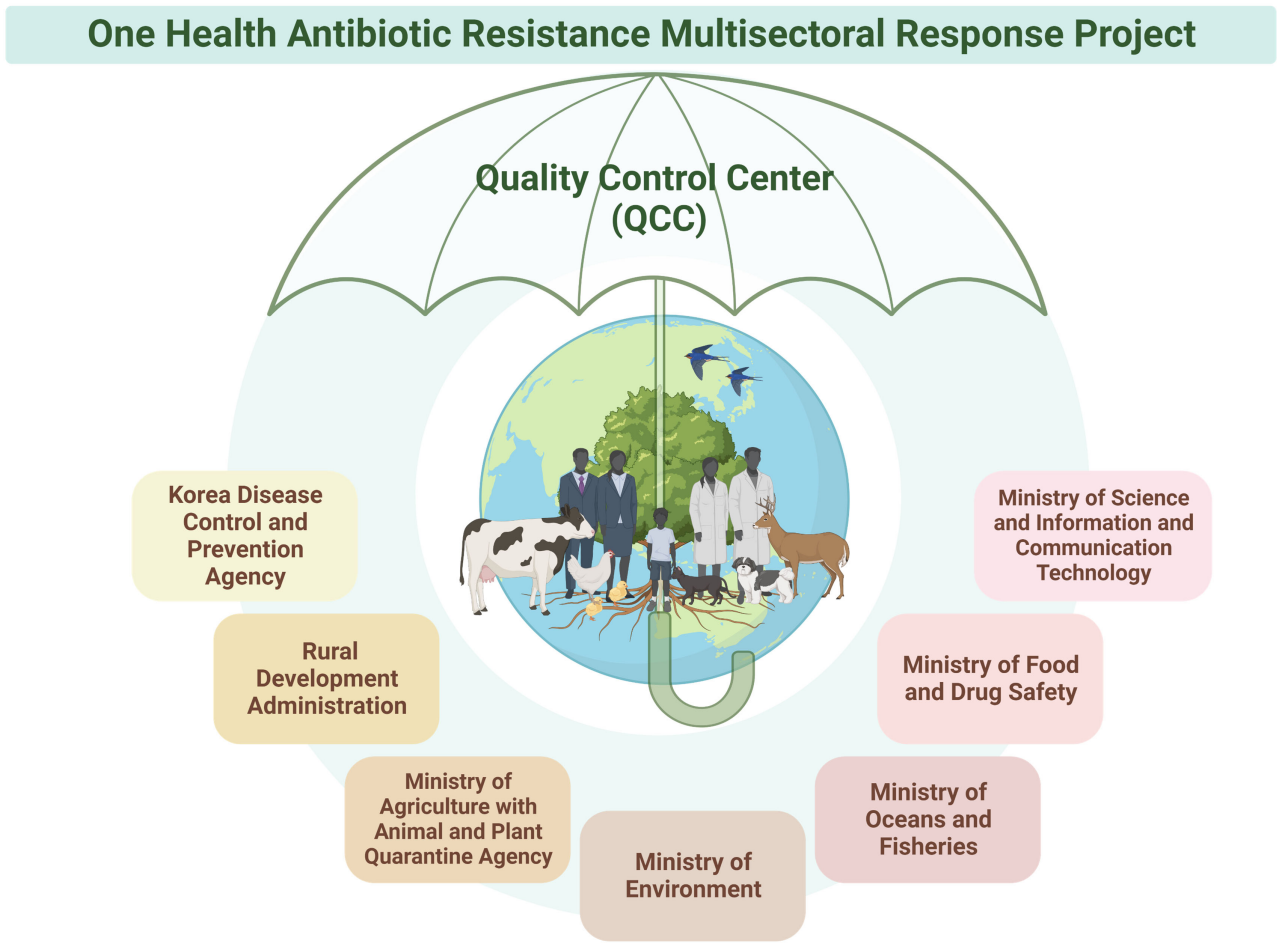
Collaboration with Seven Ministries in the One Health Antibiotic Resistance Multisectoral Response Project. The Korea Disease Control and Prevention Agency has embraced a One Health approach, forming partnerships with seven ministries. This collaborative initiative involves multidisciplinary research and development across human, animal, and environmental domains, addressing antibiotic resistance comprehensively. The project has set up an integrated network among the participating ministries to ensure coordinated management in line with research targets and objectives. Furthermore, each ministry maintains its own self-management system, with all data and management overseen by the quality control center. Figures were created with BioRender.

Antibiotics are used for humans, animals, and the environment. Consequently, bacteria with antibiotic resistance can spread across the entire ecosystem, transcending boundaries (McEwen and Collignon, 2018; Collignon and McEwen, 2019; White and Hughes, 2019). In response, Kor-Glass expanded its AMR surveillance in 2019 to encompass organisms found in humans, animals, and the environment, operating as a One Health platform to monitor resistant strains across these domains [Korea Disease Control and Prevention Agency (KDCA), 2017-2021]. Kor-GLASS has thus evolved into a comprehensive “One Health” multiagency collaborative initiative (**Figure 6**). This approach ensures seamless data sharing and collaborative efforts across the human, animal, and environmental health sectors.

The KDCA adopted a One Health approach, collaborating with seven ministries, including the Ministry of Science and ICT, Ministry of Agriculture with the Animal and Plant Quarantine Agency, Ministry of Environment, Ministry of Oceans and Fisheries, Ministry of Food and Drug Safety, and the Rural Development Administration. This collaborative initiative involves multidisciplinary research and development across human, animal, and environmental domains.

Currently, this initiative is in its early stages, undergoing a systematization process similar to Kor-GLASS, with collection centers and analysis centers being established in the animal and environmental sectors. One Health analysis centers include laboratories within regional universities, where the QCC also conducts IPT and EQA. All ministries hold quarterly working-level meetings to monitor antibiotic resistance trends and publish biannual reports discussing future policy changes and budget allocations.

## Discussion

The findings presented in this study underscore the critical importance of data quality management in AMR surveillance systems, specifically within the framework of Kor-GLASS. By expanding upon the One Health approach, Kor-GLASS has integrated data from human, animal, and environmental sectors, ensuring a comprehensive understanding of AMR trends. This integrated system, supported by a robust QCC, enables seamless data sharing and collaborative efforts among various health sectors, thereby enhancing the overall efficacy of AMR surveillance.

Kor-GLASS distinguishes itself from other national AMR surveillance systems (Bennani et al., 2021; Coombs et al., 2024; Robillard et al., 2024), through its centralized QCC. This unique feature ensures the standardization and high quality of AMR data across multiple centers. The QCC operates autonomously, providing rigorous oversight through IPT and EQA. This approach not only maintains high standards but also facilitates the rapid identification and resolution of any discrepancies or issues in the data. A recent report on the policy design, implementation, monitoring, and evaluation of national AMR action plans across 114 countries noted the absence of processes that guarantee the acquisition of standardized, high-quality data (Patel et al., 2023). This gap in systematization might explain why, even after a decade of GLASS operation, conducting a comprehensive analysis of the global AMR situation based on the available data remains challenging (Diseases, 2023).

The adoption of the One Health approach by Kor-GLASS marks a significant advancement in AMR surveillance, aligning with recent trends (Ruckert et al., 2024; Sabbatucci et al., 2024). Through this expansion, collaboration among all government departments involved in antibiotic use is ensured, enabling a comprehensive and multidisciplinary response to AMR. This collaboration facilitates the integration of diverse data sets and promotes a holistic understanding of AMR dynamics across different ecosystems.

A key strength of Kor-GLASS lies in its rigorous process and data level standardization, including the standardization of testing panels. Detailed protocols and procedures for IPT and EQA have been established and are consistently followed across all participating centers. The standardized operational framework ensures that specimen collection, data generation, and quality assessment are conducted uniformly, enhancing the reliability and comparability of the data. Additionally, the adoption of standardized data formats, ontologies, and semantics supports robust data management practices and facilitates international comparisons. The commitment to consistency and reliability is further underscored by the uniform implementation of species identification using MALDI-TOF and AST using disk diffusion methods, as detailed in **Supplementary Table S2**. This ensures that all analysis centers adhere to the same high standards.

While the client-server architecture employed by Kor-GLASS database may appear traditional, it offers several advantages, including centralized control, enhanced data security, and streamlined data quality management through the QCC. These benefits are crucial for maintaining the integrity and consistency of AMR data. The centralized approach allows for efficient oversight and rapid response to any data quality issues, ensuring that the system remains robust and reliable. Although the client-server model has limitations, such as potential scalability issues and reliance on a central point of control, these are mitigated within the Kor-GLASS framework through rigorous data validation and real-time monitoring capabilities, ensuring continuous system performance and reliability.

Since the first antibiotic was developed in the 1960s, only six new antibiotics have been introduced for clinical use (Walesch et al., 2023). As antibiotic development has reached its limits, the appropriate utilization and management of existing antibiotics are essential. Preventing AMR and protecting human health require sustainable efforts and surveillance systems. It is imperative to optimize the enhancement and utilization of GLASS data globally and prioritize overcoming this grave threat through concerted efforts across academia, commerce, public health, and policy sectors (Pallett et al., 2024). Amid slow antibiotic development and the relentless threat of superbugs, there is a need to comprehensively manage the complex interactions between humans, animals, and the environment while supporting robust data management practices. Therefore, continuous efforts to reduce AMR and strengthen global surveillance are necessary to create a sustainable future.

In conclusion, while economic and political disparities, as well as resource limitations among countries, must be taken into account when developing a global AMR management framework (Charani et al., 2023; Diseases, 2023), the enhanced functionalities of the Kor-GLASS system—particularly the integration of the One Health approach and the establishment of a centralized QCC—represent substantial advancements in the field of AMR surveillance. By addressing the critical aspects of data and process standardization, Kor-GLASS provides a model for high-quality, comprehensive AMR monitoring that can inform policy decisions and public health strategies. The ongoing collaboration among multiple ministries and the rigorous data quality management practices ensure that Kor-GLASS remains at the forefront of AMR surveillance, contributing valuable insights to the global effort to combat antimicrobial resistance.

## Data availability statement

The raw data supporting the conclusions of this article will be made available by the authors, without undue reservation.

## Ethics statement

The study protocol was approved by the Institutional Review Board of Seoul National University Bundang Hospital (no. X-2308-846-903).

## Author contributions

HyK: Data curation, Formal analysis, Validation, Visualization, Writing – original draft, Writing – review & editing. JP:Conceptualization, Data curation, Investigation, Methodology, Project administration, Validation, Writing – review & editing. DK: Project administration, Resources, Writing – review & editing. HeK: Project administration, Resources, Writing – review & editing. JeS: Project administration, Resources, Writing – review & editing. YK: Project administration, Resources, Writing – review & editing. YU: Project administration, Resources, Writing – review & editing. SK: Project administration, Resources, Writing – review & editing. JoS: Project administration, Resources, Writing – review & editing. SJ: Project administration, Resources, Writing – review & editing. KP: Conceptualization, Data curation, Funding acquisition, Investigation, Methodology, Project administration, Resources, Supervision, Validation, Writing – review & editing.
