# Supplementary figures and images for: Standardization of an antimicrobial resistance surveillance network through data management
Source: Front Cell Infect Microbiol. 2024 Jul 29;14:1411145. doi: 10.3389/fcimb.2024.1411145 (PMC11317371; doi:10.3389/fcimb.2024.1411145)

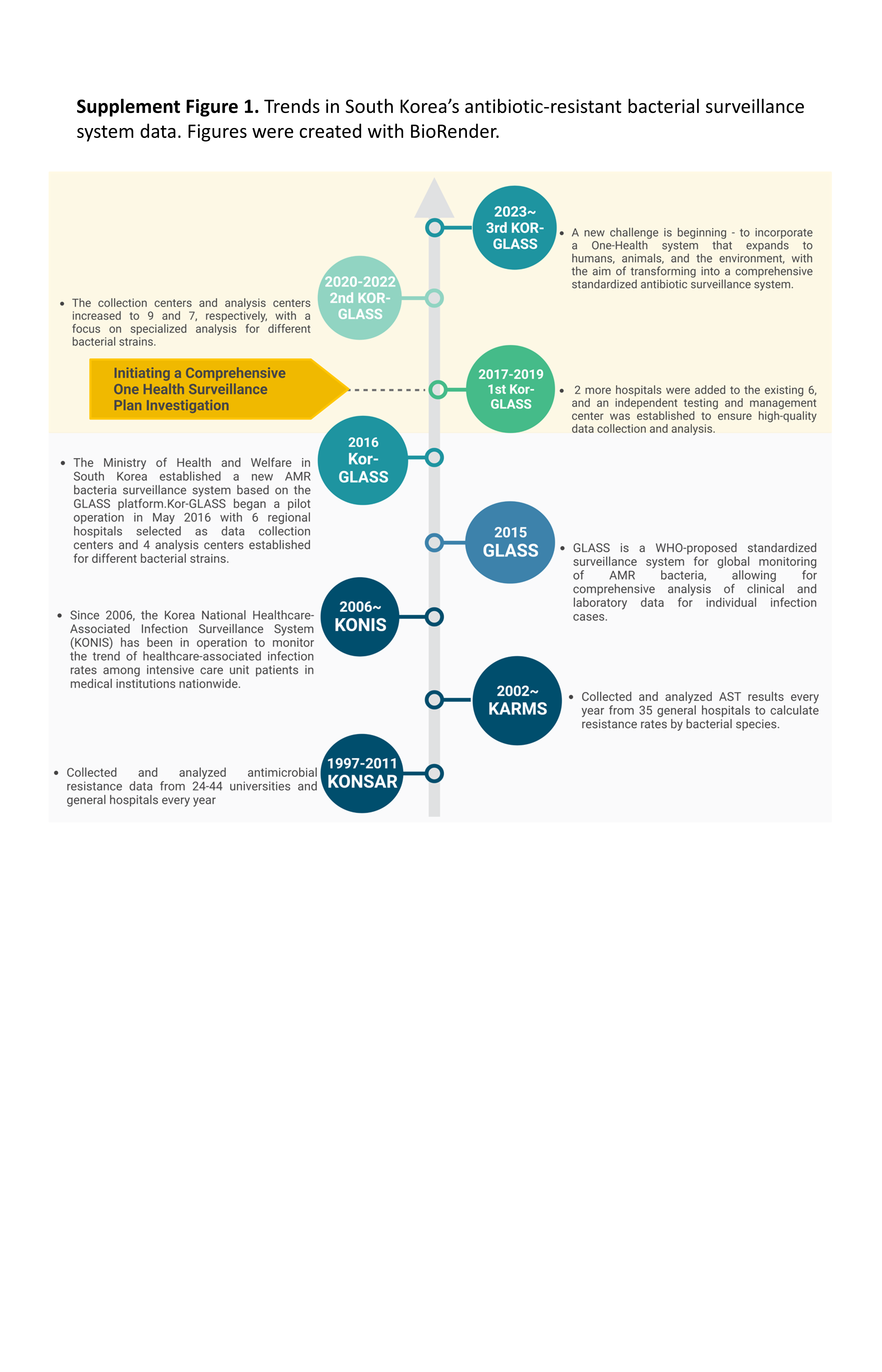

Supplement: Supplementary file 1 [file Image_1.tif]

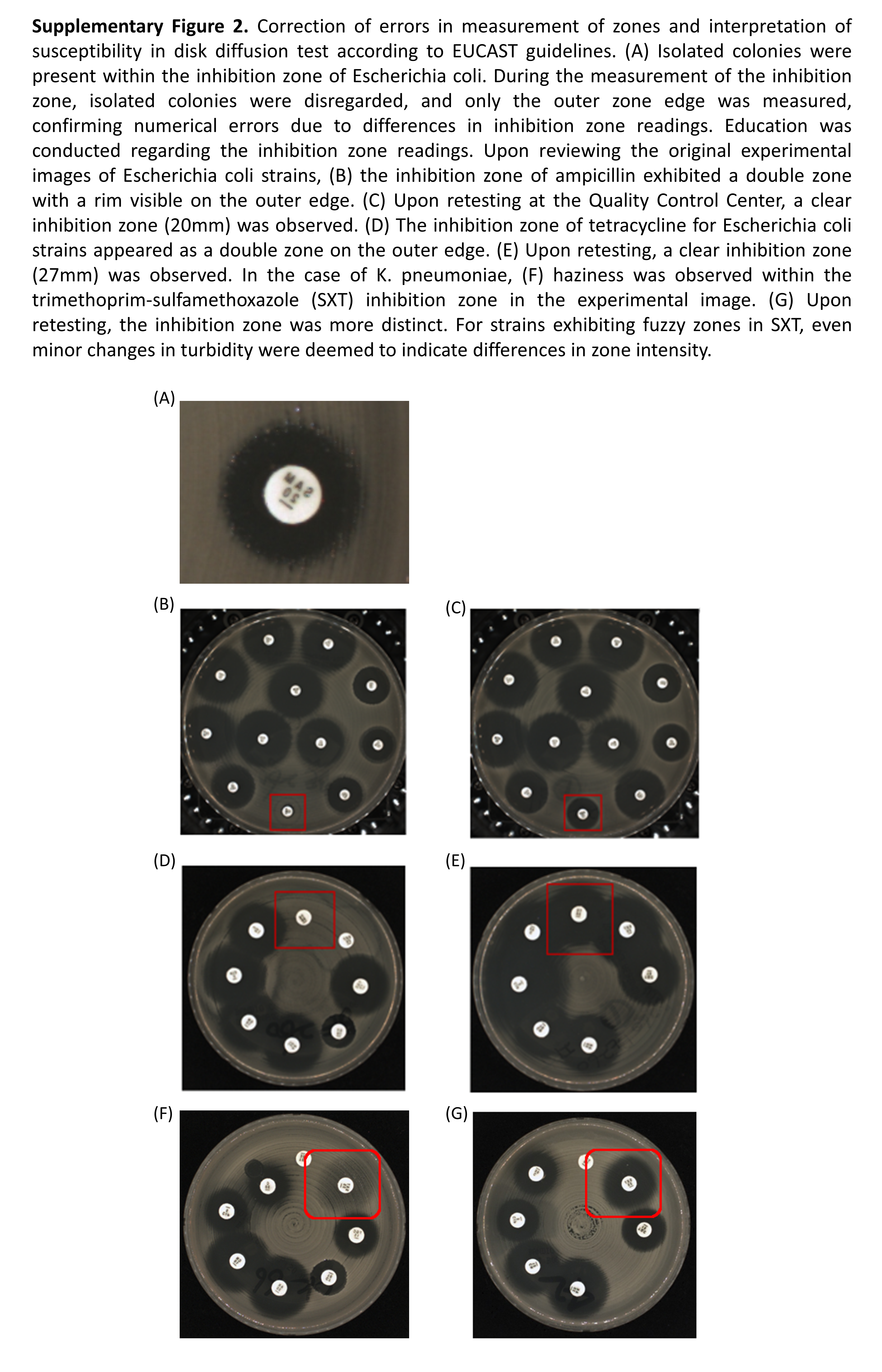

Supplement: Supplementary file 2 [file Image_2.tif]
