# Supplementary material for: Standardization of an antimicrobial resistance surveillance network through data management
Source: Front Cell Infect Microbiol. 2024 Jul 29;14:1411145. doi: 10.3389/fcimb.2024.1411145 (PMC11317371; doi:10.3389/fcimb.2024.1411145)
Supplement: Supplementary file 3 [file Table_1.docx]

**Supplementary Table 1**. Evolution of Antimicrobial Resistance Surveillance Systems in Korea

| Surveillance system | Period | Proposing entity | Participants | Target pathogens | Testing method | Objectives |
| --- | --- | --- | --- | --- | --- | --- |
| KONSAR | 1977-2011 | Universities | 24-44 Universities and general hospital | - Extended-spectrum *β-lactamase*-producing starins - cephamycin-resistant *Escherichia coli* - *Klebsiella pneumoniae* - imipenem-resistant *Pseudomonas aeruginosa* - ampicillin-resistant *Haemophilus inflluenza* - fluoroquinolone-resistant gram-negative bacilli - glycopeptide-resistant enterococci | No restrictions. | Collect routine susceptibility data |
| KARMS | 2002~ | KDCA | 35 secondary and tertiary hospitals with resident specialists in diagnostic pathology. | - *Staphylococcus aureus* - *Enterococcus faecalis* - *Enterococcus faecium* - *Streptococcus pneumoniae* - *E. coli* - *K. pneumoniae* - *P. aeruginosa* - *Acinetobacter baumannii* | Permit all types of antibiotic susceptibility testing methods | Collect data on antibiotic resistance.  Collecting major resistant strains and conducting analyses on resistance mechanisms and epidemiology. |
| KONIS | 2006~ | KDCA | Hospitals operating infection control units. | - Methicillin-resistant S. aureus - Vancomycin-resistant E. faecalis - Vancomycin-resistant E. faecium - Cefotaxime-resistant E. coli - Cefotaxime-resistant K. pneumoniae - Ciprofloxacin-resistant E. coli - Ciprofloxacin-resistant K. pneumoniae - Imipenem-resistant K. pneumoniae - Imipenem-resistant P. aeruginosa - Imipenem-resistant A. baumannii | Permit all types of antibiotic susceptibility testing methods | Operated for the purpose of managing medical-related infection issues.  Investigating occurrence rates and distribution of causative pathogens for bloodstream infections, urinary tract infections, and pneumonia. |
| Kor-GLASS | 2016~ | KDCA | Selecting hospitals with 500-1000 beds among domestic tertiary hospitals as collection centers per hub. | GLASS monitors a total of 13 bacterial species, including 9 priority pathogens and 4 domestically important pathogens:   - Gram-positive cocci: *S. aureus, S. pneumoniae, E. faecalis, E. faecium* - Gram-negative bacilli: *E. coli, K. pneumoniae, Salmonella spp., Shigella spp., A. baumannii, P. aeruginosa* - Gram-negative cocci: Neisseria gonorrhoeae - Anaerobic Gram-positive bacilli: *Clostridioides difficile* - Yeast-like fungi: *Candida spp* | The collection centers collect all clinical isolates of target bacterial species and deliver them to the analysis centers for strain-specific analysis.  Implemented uniformly across analysis centers. | An antibiotic resistance bacterial surveillance system overcoming limitations such as the diversity of AST testing methods and interpretation criteria among laboratories, incomplete validation of repeatedly isolated strains, and discontinuity in testing periods. |
| One Health | 2017~ | KDCA | Expansion of Kor-GLASS.  Involving 7 ministries^†^ | Expanding Kor-GLASS into the non-human sector. | Expanding Kor-GLASS into the non-human sector. | Expanding Kor-GLASS into the non-human sector. |

^†^Seven ministries include the KDCA, Ministry of Science and ICT, Ministry of Agriculture, along with the Animal and Plant Quarantine Agency, Ministry of Environment, Ministry of Oceans and Fisheries, Ministry of Food and Drug Safety, and Rural Development Administration.

- Abbreviations: KONSAR, Korean Nationwide Surveillance of Antimicrobial Resistance; KARMS, Korean Antimicrobial Resistance Monitoring System; KDCA, Korea Disease Control and Prevention Agency; KONIS, Korean Nosocomial Infections Surveillance System; GLASS, Global Antimicrobial Resistance and Use Surveillance System.

**Supplementary Table 2**. Standardized Operational Framework Process for Each of the Stages of Kor-GLASS

| No. | Center Names by Stage | Center Designation Criteria | No. of Participating Institutions | Summary of Standardized Procedures |
| --- | --- | --- | --- | --- |
| 1 | **Collection center** | To establish representativeness, hospitals with 500-1000 beds among domestic general hospitals are selected as collection centers per hub. | 9 | - The collection center collects specified bacterial specimens and clinical information and conducts pathogen isolation and species identification. In accordance with the WHO GLASS guidelines, priority pathogens are designated for each specimen type. A comprehensive investigation is conducted on target pathogens isolated from specimens such as blood, urine, feces, urethral or cervical smears, respiratory samples, and cerebrospinal fluid, excluding duplicate strains. - The collection center also verifies designated strains through subculturing for strain isolation and species identification using MALDI-TOF MS. - The clinical information collected by the center comprises 28 items, with the recognition of collected strains contingent upon the inclusion of the following 13 mandatory items:   - - Mandatory items: strain management number, birth year, birth month, gender, visit/admission date, visit type, transfer status, origin, ICU status, specimen name, specimen collection date, species, species (genus, species). - Clinical information must be uploaded to the Kor-GLASS database (https://is.kdca.go.kr/) at least one day before the strain shipping date. Specimen information is individually entered with a number composed of region-year-strain abbreviation-collection number. - Strains are shipped to the analysis center twice a month in two vials of skim milk via the designated strain transport company. This information is entered into the Kor-GLASS database, allowing the tracking of specimen flow within the database. This information is entered into the Kor-GLASS database, allowing the tracking of specimen flow within the database. - The collection center establishes an operational committee that conducts regular reviews of the overall project four times a year and reports the results to the NCC and NRL. Additionally, the collection center forms an advisory committee composed of experts from academia, industry, and research institutes. |
| 2 | **Analysis center** | To ensure specialization, general hospitals with dedicated on-site microbiology expert and certification for excellence from the Korean Foundation for Laboratory Accreditation are selected as analysis centers. | 7 | - The analysis centers are specialized for different species, and they perform species identification, AST, and resistance gene tests on the collected strains. Species identification is conducted using MALDI-TOF MS, and if necessary, 16S rRNA or *rpoB* gene sequencing is used for confirmation. Antimicrobial susceptibility testing follows CLSI and EUCAST recommendations, using disk diffusion or broth microdilution methods. - Test results are organized per strain according to the specified data format in the Kor-GLASS database. Collected information includes antibiotic test data (inhibition zone (mm), RIS, MIC), and gene test data (resistance genes, toxin genes, genotype analysis results, etc.). - Strains that have completed testing are shipped once a month to the test QCC in skim milk stock form for test management. Subsequently, strains with completed test management (2 vials per strain: 1 skim milk, 1 microbank) are sent to NRL. - An Analysis Center Research Council is established, which meets quarterly to report on domestic and international status of species, analyze resistance trends, and discuss ways to enhance species-specific expertise. The results discussed by the Analysis Center Research Council are reported to NCC and NRL. |
| 3 | **Quality control center** | To ensure specialization, selected general hospitals must have dedicated on-site microbiology experts, certification for excellence from the Korean Foundation for Laboratory Accreditation, and ISO 9001:2015 certification. | 1 | - Laboratory quality control is conducted at both the collection and analysis centers. Regular IPT (Internal Proficiency Testing) and EQA (External Quality Assessment) are performed (refer to the main text for methods). IPT results, EQA result reports, and action outcomes are reported to the Kor-GLASS database.   - Collected information includes antibiotic test data (inhibition zone (mm), RIS, MIC) and gene test data (resistance genes, toxin genes, genotype analysis results, etc.). - The results of test management are utilized to improve the quality of education. The QCC also provides training on testing methods to researchers at participating institutions and assists in resolving testing issues. - The QCC conducts separate test management to stabilize the testing capabilities of each institution when new institutions participate or when testing methods change. - The QCC develops and manages reference strains for test management and provides them to each institution. |
| 4 | **KDCA** | NCC and NRL | 1 | - The Kor-GLASS DB is centrally managed. The roles are divided as follows:   - NCC Role: Perform project planning and management.   - NRL Role: Create and distribute standard testing manuals, provide laboratory technical support and quality control assistance, analyze and generate statistical data, manage the antibiotic resistance resource and information database, and identify major resistant strains and trends. - The national antibiotic resistance rates derived from the Kor-GLASS database are reported annually to GLASS. These results are made available to other stakeholders and the general public through a site linked to the KDCA website (https://nih.go.kr/nohas/common/main.do), where national statistics are published. |

- Abbreviations: WHO, World Health Organization; GLASS, Global Antimicrobial Resistance and Use Surveillance System; ICU, Intensive Care Unit; MALDI-TOF MS, matrix-assisted laser desorption/ionization time-of-flight mass spectrometry; NCC, National Coordinating Center; NRL, National Reference Laboratory; AST, antimicrobial susceptibility testing; CLSI, Clinical Laboratory Standards Institute; EUCAST, European Committee on Antimicrobial Susceptibility Testing; RIS, resistant-intermediate-susceptible; IPT, interlaboratory proficiency testing; EQA, external quality assessment; KDCA, Korea Disease Control and Prevention Agency.
